# Cu_2_O Photocathode for Low Bias Photoelectrochemical Water Splitting Enabled by NiFe-Layered Double Hydroxide Co-Catalyst

**DOI:** 10.1038/srep30882

**Published:** 2016-08-04

**Authors:** Huan Qi, Jonathan Wolfe, Denis Fichou, Zhong Chen

**Affiliations:** 1 School of Materials Science Engineering, Nanyang Technological University, Singapore 639798, Singapore; 2 Interdisciplinary Graduate School, Nanyang Technological University, Singapore 639798, Singapore; 3 School of Physical and Mathematical Sciences, Nanyang Technological University, 637371, Singapore; 4 CNRS, UMR 8232, Institut Parisien de Chimie Moléculaire, Paris, France; 5 Sorbonne Universités, UPMC Univ Paris 06, UMR 8232, Institut Parisien de Chimie Moléculaire, F-75005, Paris, France

## Abstract

Layered double hydroxides (LDHs) are bimetallic hydroxides that currently attract considerable attention as co-catalysts in photoelectrochemical (PEC) systems in view of water splitting under solar light. A wide spectrum of LDHs can be easily prepared on demand by tuning their chemical composition and structural morphology. We describe here the electrochemical growth of NiFe-LDH overlayers on Cu_2_O electrodes and study their PEC behavior. By using the modified Cu_2_O/NiFe-LDH electrodes we observe a remarkable seven-fold increase of the photocurrent intensity under an applied voltage as low as −0.2 V *vs* Ag/AgCl. The origin of such a pronounced effect is the improved electron transfer towards the electrolyte brought by the NiFe-LDH overlayer due to an appropriate energy level alignment. Long-term photostability tests reveal that Cu_2_O/NiFe-LDH photocathodes show no photocurrent loss after 40 hours of operation under light at −0.2 V *vs* Ag/AgCl low bias condition. These improved performances make Cu_2_O/NiFe-LDH a suitable photocathode material for low voltage H_2_ production. Indeed, after 8 hours of H_2_ production under −0.2 V *vs* Ag/AgCl the PEC cell delivers a 78% faradaic efficiency. This unprecedented use of Cu_2_O/NiFe-LDH as an efficient photocathode opens new perspectives in view of low biasd or self-biased PEC water splitting under sunlight illumination.

The accelerated depletion of fossil fuel reserves combined with increasing demand of energy across the world has triggered a tremendous effort toward alternative energy sources and various technologies are currently being explored. Among those, photoelectrochemical (PEC) water splitting under solar light irradiation has shown their potential by the successful integration of abundant energy source and high efficient catalysts. In order to realize high efficiency PEC systems in view of water splitting, a number of photocatalysts have been investigated including the widespread TiO_2_^1^,^2^,^3^, ZnO^4^,^5^,^6^,^7^, WO_3_^8^,^9^,^10^,^11^, Fe_2_O_3_^12^, as well as more innovative catalysts such as Cu_2_O^13^,^14^,^15^, and BiVO_4_^16^.

Cu_2_O is a promising photocathode material due to its suitable band gap (1.9–2.2 eV) and high absorption coefficient in the visible region^17^,^18^,^19^,^20^. Its conduction band is located above the reduction potential of water, making it suitable for mediating H_2_ production with little or no external bias in a PEC cell. Among different methods, electrodeposition has proven to be the most convenient and reliable to prepare nanostructured Cu_2_O^17^,^21^. Recent studies show that the Cu_2_O morphology and orientation can be controlled by judiciously tuning the deposition parameters such as the electrolyte pH, temperature, applied potential and current density^17^,^21^. The morphology and crystal structure determine the material performances through their role in light absorption and charge transport.

Although Cu_2_O possesses an intrinsic potential as a photocathode, the reported photocurrents remain well below the theoretical values, in particular under low external biases. These poor performances arise essentially from structural defects at the semiconductor-electrolyte interface. It has been reported that at low external voltages, Cu_2_O does not transfer electrons efficiently towards the electrolyte due to an inappropriate band bending^22^. In order to improve the photocurrent of Cu_2_O electrodes, various approaches have been recently developed. For example Cu_2_O can be combined with another catalyst such as TiO_2_^23^, or by depositing a protective layer on its surface such as Cu_2_S^13^,^14^, RuO_2_^24^, or polyoxometallates^25^.

Layered double hydroxides (LDHs) attract a growing attention as co-catalysts due to several advantages. LDHs constitute a group of two-dimensional materials of general formula [M_1−x_^2+^M_x_^3+^(OH)_2_][A_x/n_]·mH_2_O in which a fraction of a divalent metal cation M^2+^ coordinated octahedrally by hydroxy groups is replaced isomorphously by a trivalent metal cation M^3+ ^26,27. The variety in chemical composition and structural morphology of LDH materials make them suitable for a wide range of applications as electrocatalysts^28^,^29^,^30^,^31^,^32^. In particular, LDHs can act as efficient photocatalysts for improving the charge separation of photogenerated electrons and holes^33^. Moreover, the hierarchical morphology of LDHs provides convenient charge transfer at the electrolyte interface in PEC systems. Several types of LDHs such as CoNi^33^, or ZnCo^34^, have been widely investigated to enhance PEC water splitting.

We describe here the fabrication of Cu_2_O/NiFe-LDH via a facile two-step electrodeposition method and the use of NiFe-LDH to enhance the performances of Cu_2_O photocathodes. The NiFe-LDH layers grow as nanoplatelets that are uniformly anchored onto the Cu_2_O surface. Our Cu_2_O/NiFe-LDH exhibits greatly improved photocurrent intensities particularly at low applied voltages. Moreover we demonstrate that modified Cu_2_O/NiFe-LDH photocathodes allow more efficient H_2_ production at low applied voltages. The Cu_2_O/NiFe-LDH photocathodes reveal to be highly stable with no degradation under low bias after 40 hours of illumination, making Cu_2_O/NiFe-LDH an excellent photoelectrode for low bias PEC water splitting.

## Results and Discussion

### Morphology of Cu_2_O/NiFe-LDH materials

**Figure 1:**
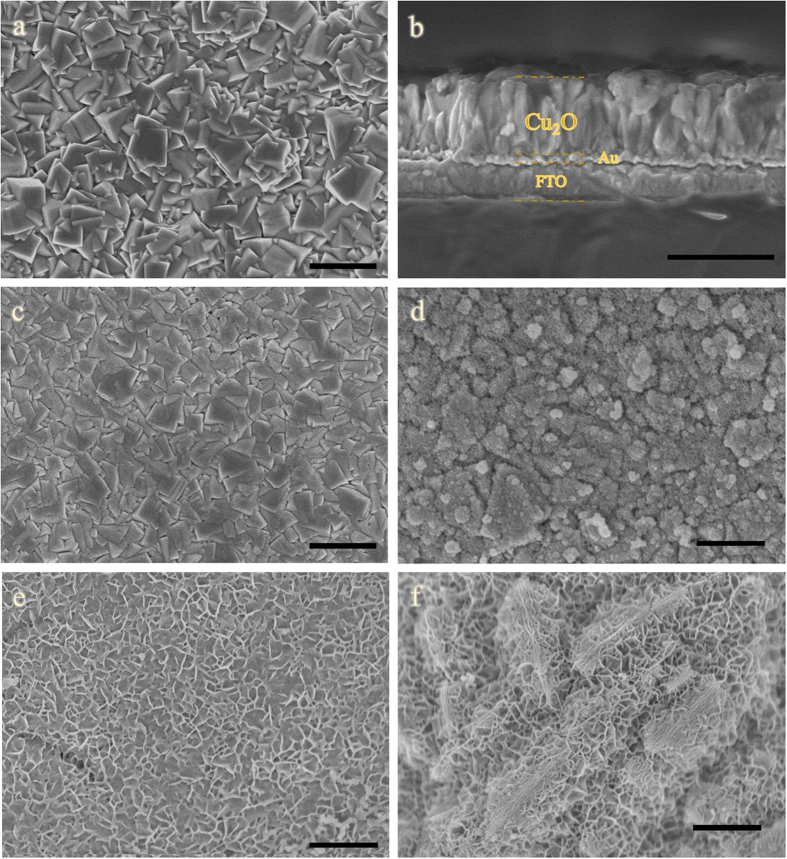
FESEM images of (**a**) bare Cu_2_O; (**b**) cross-sectional image of bare Cu_2_O; (**c**) Cu_2_O/NiFe-LDH (20 s); (**d**) Cu_2_O/NiFe-LDH (60 s); (**e**) Cu_2_O/NiFe-LDH (90 s) and (**f**) Cu_2_O/NiFe-LDH (300 s). Scale bars: 1 μm.

Images recorded by field-emission scanning electron microscopy (FESEM) show that electrodeposited Cu_2_O consists of compact and highly homogeneous layers made of cubic particles having ~400 nm in size (Fig. 1a) and thicknesses of ~1 μm after 1,500 s of deposition time (Fig. 1b). NiFe-LDH overlayers are then grown on the Cu_2_O samples using different deposition times (Fig. 1b,c). After 60 seconds, the NiFe-LDH material adopts a uniform nanoflake morphology (Fig. 1d) with individual flakes of size ~5 nm (Figure S1). After 300 s it self-assembles into sponge-like structures (Fig. 1f). AFM images show that after deposition of NiFe-LDH, the surface roughness is greatly increased (Table S1 and Figure S2) and may thus increase substantially the number of photons absorbed by the photocathode. Besides, the XRD pattern of NiFe-LDH after 150 s exhibits typical (003), (006), and (104) reflection peaks, while Cu_2_O shows the known dominant (111) reflection (Figure S3)^35^,^36^. For NiFe-LDH (20 s) XRD does not reveal any visible peaks, while EDX images confirm the existence and uniformity of Ni and Fe elements in the Cu_2_O layer (Figure S4).

### PEC measurement

#### Linear sweep voltammetry

**Figure 2:**
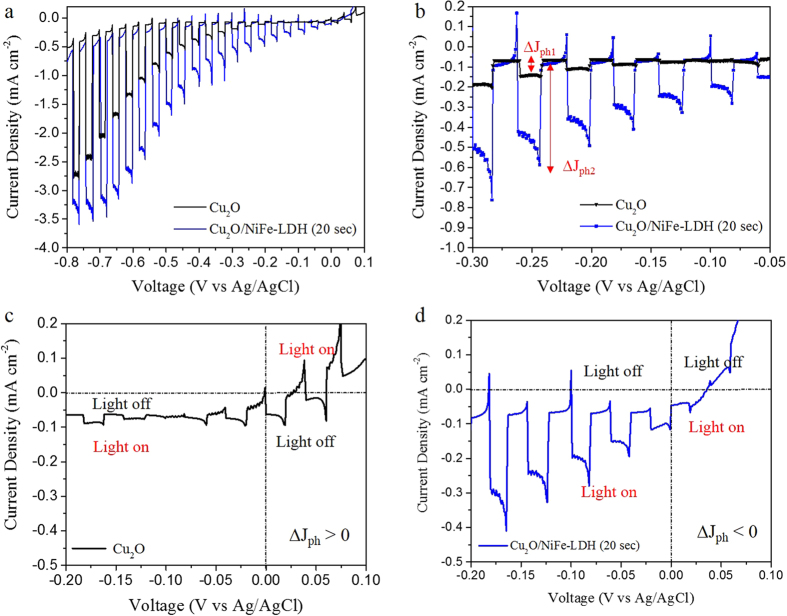
Linear sweep voltammetry under light-chopped illumination of bare Cu_2_O and Cu_2_O/NiFe-LDH (20 s) electrodes in the voltage ranges (**a**) +0.1/−0.8 V and (**b**) −0.3/−0.05 V vs Ag/AgCl. ∆J_ph_ denotes the photocurrent density after substraction of the dark current. At ‒0.25 V vs Ag/AgCl, the ∆J_ph_ value for bare Cu_2_O is ∆J_ph1_ = 0.07 mA.cm^−2^ while for Cu_2_O/NiFe-LDH it is ∆J_ph2_ = 0.49 mA.cm^−2^. (**c,d**) Zoom-in of the photoresponse of Cu_2_O and Cu_2_O/NiFe-LDH electrodes in the −0.2/+0.1 V vs Ag/AgCl low voltage range.

The PEC properties of Cu_2_O with and without NiFe-LDH were investigated under chopped light by linear sweep voltammetry (LSV) (Fig. 2a and Figure S5a). Before deposition of NiFe-LDH the photocurrent density is 1.27 mA.cm^−2^ under −0.6 V *vs* Ag/AgCl. For deposition times shorter than 20 s the photocurrent of bare Cu_2_O does not improve significantly (Figure S6). After deposition of NiFe-LDH for 20 s, the photocurrent density exhibits a twofold increase to reach 2.42 mA.cm^−2^. An even more important relative photocurrent increase is observed in the low bias range from 0.0 V to −0.3 V *vs* Ag/AgCl. For example, Cu_2_O/NiFe-LDH (20 s) under −0.2 V *vs* Ag/AgCl, that is more than seven times that of bare Cu_2_O (−0.05 mA.cm^−2^, Fig. 2b). It shows that the introduction of the NiFe-LDH layer induces a more efficient separation of photogenerated charges on one side and an effective electron injection into the electrolyte on the other side. Prolonged deposition of NiFe (more than 20 s) results in less or no improvement in photocurrent (Figure S5a) due to a too strong light absorption by NiFe-LDH that reduces charge carriers generation by Cu_2_O.

Furthermore, although bare Cu_2_O generates a *positive* photocurrent when illuminated under *positive* low voltages (Fig. 2c), the Cu_2_O/NiFe-LDH electrodes generates a *negative* photocurrent under *negative* voltages (Fig. 2d and Figure S5c,d). For example, under −0.1V vs Ag/AgCl, Cu2O electrode produces a very weak *positive* photocurrent (< + 0.01 mA.cm^−2^) while Cu_2_O/NiFe-LDH (20 s) generates a *negative* photocurrent (−0.3 mA.cm^−2^). Note that for reversed voltages (that is negative for bare Cu_2_O and positive for Cu_2_O/NiFe-LDH) both types of electrodes do not produce any significant photocurrent. Whereas, this photocurrent reversal is another evidence of the profound modification brought by the NiFe-LDH (20 s) overlayer on the PEC behaviour of Cu_2_O electrodes.

The Mott-Schottky plots comparison of Cu_2_O photocathode with and without NiFe-LDH (20 s) reveals the greatly increased number of charge carriers that has reached the interface is induced by NiFe-LDH cocatalyst (Figure S7). This unveils the function of NiFe-LDH as an efficient cocatalyst that improves the charge separation of Cu_2_O photocathode. Furthermore, in order to eliminate the possibilities that this photocurrent enhancement is induced by the metal valence state changes in NiFe-LDH, we have investigated the cyclic voltammetry behaviour of NiFe-LDH (Figure S8). Results indicate that at the low bias condition (less than −0.2 V *vs* Ag/AgCl), there is no reduction/oxidation behaviour on NiFe-LDH. This excludes the likelihoods of metal valence state changes-induced photocurrent enhancement of Cu_2_O/NiFe-LDH photocathode in low bias condition. The photoresponse of pure NiFe-LDH (300 s) has also been explored (Figure S9). Results show that NiFe-LDH itself generates very limited charge carrier upon illumination. This excludes the possibilities that the photocurrent enhancement of Cu_2_O/NiFe-LDH photocathode is induced by the increased number of photon-generated charge carriers induced by NiFe-LDH.

Another point worth notice is that the difference in photocurrent of Cu_2_O photocathode and Cu_2_O/NiFe-LDH photocathode becomes smaller with increased bias voltage. This is because the increased high applied voltage is able to efficiently separate the majority of photon-generated charge carriers. The effects of NiFe-LDH become less pronounced. As there is no difference for the total number of charge carriers as Cu_2_O is the only photon absorber (Figure S9). Thus, the maximum photocurrent of both electrodes have very little difference.

#### Electrochemical impedance spectroscopy

**Figure 3:**
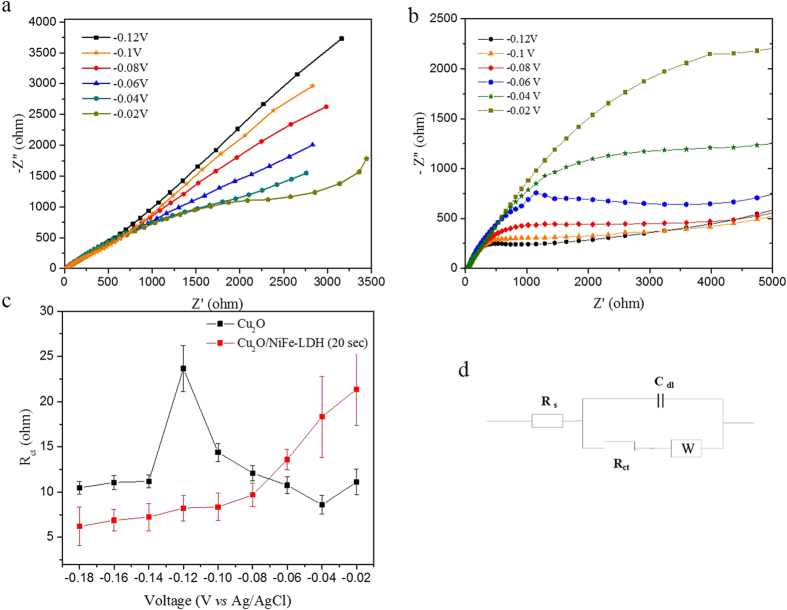
Electrochemical impedance spectroscopy of (**a**) Cu_2_O and (**b**) Cu_2_O/NiFe-LDH (20 s) electrodes under different external biases. (**c**) Voltage dependence of the resistance R_ct_ of Cu_2_O and Cu_2_O/NiFe-LDH electrodes using the equivalent circuit shown in (**d**).

Electrochemical impedance spectroscopy (EIS) provides information about the interfacial property of the synthesized photocathode, which further reveals the efficient charge separation effect and improved electron injection property brought by NiFe-LDH layer (Fig. 3). The semi-circular diameter of the measured EIS stands for the charge carrier transfer resistance (R_ct_) that controls the electron transfer kinetics at the electrode/electrolyte interface^37^. The resistance of Cu_2_O/NiFe-LDH in the dark is much larger than that under illumination, indicating a higher number of charge carriers at the electrode interface. (Figure S10). In addition, as compared with bare Cu_2_O, the radius of the semicircle of Cu_2_O/NiFe-LDH is smaller under all the conditions (Fig. 3a,b and Figure S11).

**Figure 4:**
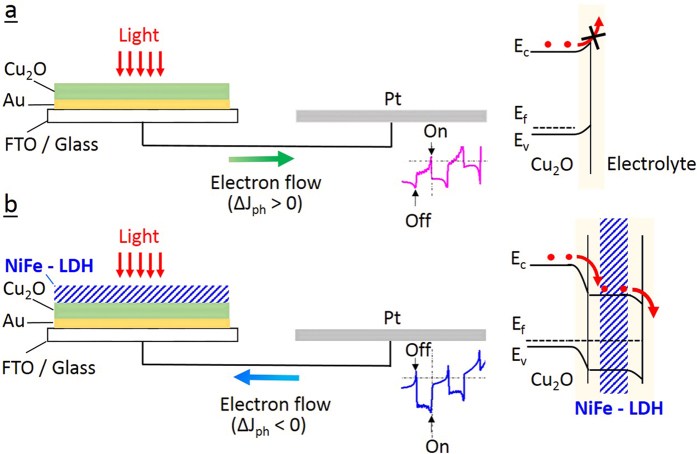
Schematics of photogenerated electron transfer occurring in a PEC system using (**a**) bare Cu_2_O and (**b**) modified Cu_2_O/NiFe-LDH (20 s) electrodes. The reversal of the electron flow at low voltages is indicated as well as the corresponding band diagrams (red dots denote electrons).

An interesting observation is that by increasing the applied bias from −0.02 V to −0.12 V *vs* Ag/AgCl, the interface resistance increases for bare Cu_2_O but decreases for the Cu_2_O/NiFe-LDH samples. This phenomenon arises from the difference between the electrode/electrolyte interfaces with either Cu_2_O or NiFe-LDH. Under illumination over the voltage range −0.02/−0.12 V vs Ag/AgCl, the surface of bare Cu_2_O is predominantly charged with holes essentially because of the blockage of the electron transfer path from Cu_2_O to the electrolyte (see Fig. 4a). At more negative voltages, the number of holes decreases, thus resulting in a resistance increase of bare Cu_2_O. This is evidenced by the larger semi-circle diameter in the EIS (Fig. 3a). In contrast, deposition of a NiFe-LDH overlayer induces an appropriate energy level alignment with respect to the electrolyte redox levels so that electrons are efficiently transported towards the surface where they reduce water into H_2_ (Fig. 4b and Figure S12b). Electrons become predominant and the NiFe-LDH surface is negatively charged. The number of electrons increases with increasing negative voltage, as confirmed by the smaller semi-circle diameter in Fig. 3b. This demonstrates the key role of NiFe-LDH in introducing appropriate energy levels at the interface and the subsequent higher electron injection rate into the electrolyte.

Overall, the inefficient surface charge separation efficiencies and insufficient electron transfer in the electrolyte of bare Cu_2_O induces an overwhelming number of photogenerated electrons in the bulk. On the contrary, after coating by NiFe-LDH, electrons are able to transfer from electrode to electrolyte under very low external bias (Fig. 4). This results in a reduced number of electrons in the Cu_2_O layer, driving electrons from the counter-electrode to the Cu_2_O photocathode. As a result, the sample with and without NiFe-LDH have different electron flow directions for a given bias (Fig. 4 and Figure S12). This superior property makes Cu_2_O/NiFe-LDH an excellent candidate for photocathode in low or non-bias PEC systems.

### Photostability

**Figure 5:**
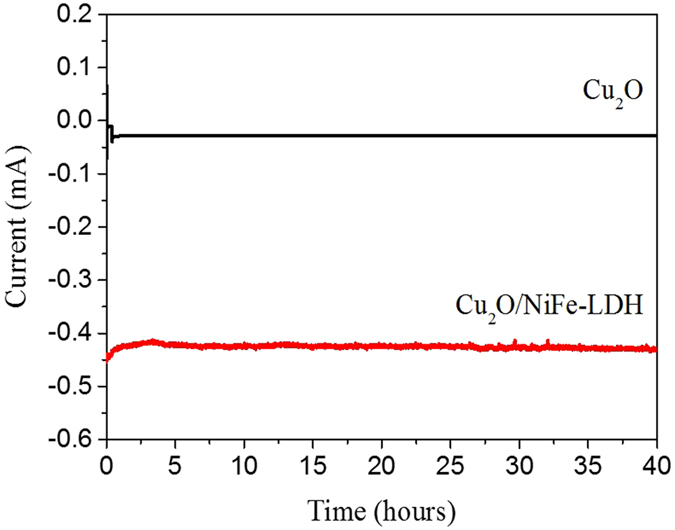
Long-term photostability of bare Cu_2_O (black curve) and Cu_2_O/NiFe-LDH (20 s) (red curve) electrodes under white light illumination and −0.2 V vs Ag/AgCl external voltage.

In order to evaluate the potential of Cu_2_O/NiFe-LDH photocathodes for H_2_ production, we tested their long-term stability under illumination at low voltages. It is well-known that Cu_2_O suffers from poor stability under illumination. The photogenerated electrons reduce Cu_2_O into Cu and photogenerated holes oxidize Cu_2_O into CuO. When oxidized, the illuminated area rapidly turning black. Various attempts have been made to solve this problem^38^,^39^,^40^,^41^, but the stability of Cu_2_O under low bias is rarely mentioned because of too low photocurrents. However, in the presence of the NiFe-LDH co-catalyst, the photostability of Cu_2_O under low bias can be explored. Observations indicate that Cu_2_O instability is mostly caused by the highly negative applied voltage, under which electrons that accumulate at the photocathode reduce Cu_2_O into metallic copper (Figure S13). The testing conditions in the literature are usually under ~0V *vs* RHE^17^, under which environment Cu_2_O tends to be reduced to a most stable state – metallic Cu. Whereas, our testing condition is −0.2 V *vs* Ag/AgCl under pH 6.5, which fell exactly in its stable condition range. This is supported by the fact that Cu_2_O samples under −0.2 V *vs* Ag/AgCl is also surprisingly stable after 40 hours of continuous illumination (Fig. 5). The stability tests of Cu_2_O with and without NiFe-LDH under −0.6 V *vs* Ag/AgCl show that NiFe-LDH also lengthens the stability of Cu_2_O under relatively high voltage (Figure S14). Whereas, the purpose of this study to improve Cu_2_O photocathode’s performance under its stable condition (green shadowed area in Figure S13) instead of spend numerous efforts in improving its stability under extreme conditions such as under strong negative bias.

### H_2_ production

**Figure 6:**
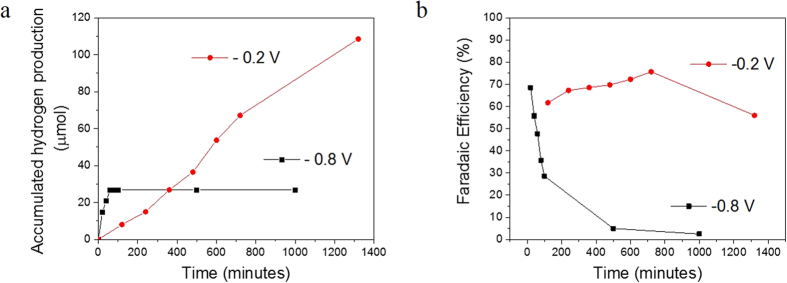
(**a**) Hydrogen production and (**b**) faradaic efficiency of a PEC system using Cu_2_O/NiFe-LDH (20 s) as the working electrode under applied voltages of −0.2 V vs Ag/AgCl (red curves) and −0.8 V vs Ag/AgCl (black curves).

H_2_ evolution tests using Cu_2_O/NiFe-LDH (20 s) photocathodes have been conducted in a 25% methanol solution as a sacrificial reagent under visible light irradiation according to literature^42^. The faradaic efficiency is calculated according to ρ = n_H2_/(Q/2F), where n_H2_ is the amount of hydrogen generated, Q is the total amount of charge passed through the cell (C), and F is the faraday constant. As shown in Fig. 6, under low bias −0.2 V *vs* Ag/AgCl, the initial faradaic efficiency is 61%, and it increases with time up to 78% before decreasing slowly after 800 minutes of illumination. There is no significant decrease in the performance during the first 12 hours. Over the following 10 hours the efficiency slightly decreases to 59%. When a higher bias of −0.8 V *vs* Ag/AgCl is applied, it shows good H_2_ evolution in the first 40 minutes. However, after 60 minutes H_2_ stops evolving and a progressive decrease of the faradaic efficiency takes place to become less than 5% after 1,000 minutes of illumination.

## Conclusions

The growth of an ultrathin NiFe-LDH co-catalyst on Cu_2_O electrodes by electrodeposition induces a remarkable seven-fold increase of the photocurrent density under low applied voltages (typically 0.49 mA.cm^−2^ at ‒0.25 V *vs* Ag/AgCl, as compared to 0.07 mA.cm^−2^ for bare Cu_2_O). Electrochemical impedance spectroscopy reveals that NiFe-LDH (20 s) considerably reduces the Cu_2_O surface resistance thus allowing photogenerated electrons to be efficiently transported and injected into the electrolyte. Both results are induced by the appropriate band alignment induced by NiFe-LDH. When coated with NiFe-LDH (20 s) as a co-catalyst, the Cu_2_O photocathodes generate negative photocurrents with an excellent stability over 40 hours of continuous visible illumination under an external bias of –0.2 V *vs* Ag/AgCl. In Cu_2_O/NiFe-LDH (20 s) photocathodes, the combination of high photocurrents and long-term stability under low voltage allow hydrogen evolution. After 8 hours of continuous illumination, Cu_2_O/NiFe-LDH photocathodes exhibit a 78% Faradaic efficiency under –0.2 V *vs* Ag/AgCl, while the efficiency drops down to only 5% under −0.8 V *vs* Ag/AgCl. These results demonstrate that modified Cu_2_O/NiFe-LDH photoelectrodes are well-adapted to low-biased or self-biased PEC systems in view of artificial photosynthesis.

## Methods

### Preparation of Cu_2_O photocathodes

Prior to the deposition, 50 nm Au was deposited on FTO glasses via e-beam deposition to ensure the conductivity and reproducibility of the samples. Electrodeposition of Cu_2_O was carried out in a basic solution of lactate-stabilized copper sulfate consisting in 0.2 M CuSO_4_ (Sigma Aldrich) and 3 M lactic acid (Fisher Scientific) solution in deionized water. Afterward, 1 M NaOH was added to adjust the solution pH to 12. The basic environment ensures that deposited Cu_2_O is of p-type conduction. During deposition, the temperature was kept constant at 40 °C using a hot plate. The Cu_2_O thin films were deposited at a constant current density of −1 mA·cm^−2^ *vs* Ag/AgCl reference electrode using a three-electrode system (galvanostatic mode, Gamry Instruments, Inc.)

### Electrodeposition of the NiFe-LDH co-catalyst

Electrodeposition of NiFe-LDH is realized using Cu_2_O as the working electrode, together with a Pt counter-electrode and Ag/AgCl as the reference electrode. The solution for electrodeposition of NiFe-LDH consists in a mixture of 0.2 M [Ni(NO_3_)_2_∙6H_2_O] and 0.1 M [Fe(NO_3_)_3_∙9H_2_O] in 50 ml of deionized water. A constant potential of −1.0 V *vs* Ag/AgCl is applied during various periods of time in the range 20–300 s, leading to different morphologies. For less than 60 s, NiFe-LDH does not reveal a clear nanoflake morphology, although photocurrent is already improved as compared with bare Cu_2_O. A 150 s deposition time is required to obtain visible XRD peaks due to the intensity limit.

### Morphological characterizations

The crystal phase of the synthesized photocathodes were studied using a Shimadzu thin film X-ray diffractometer with a Cu Ka excitation (λ = 1.54 Å). Microscopic morphologies including lattice analysis of scraped particles were obtained using field emission scanning electron microscopy (FESEM, JEOL JSM-7600F) and high resolution transmission electron microscopy (HRTEM, JEOL-2100F) operating at 200 kV.

### Photoelectrochemical measurements

All PEC measurements were conducted in a 0.5 M Na_2_SO_4_ electrolyte using a three-electrode configuration with synthesized sample as the working electrodes, Pt and Ag/AgCl electrodes being used as the counter and reference electrodes, respectively. The inter-electrode spacing was ~1 cm. Photocurrents were recorded using a PCI4/300™ potentiostat equipped with PHE200™ software (Gamry Instruments, Inc.). The working electrodes were exposed to the AM 1.5 light from a solar simulator equipped with a 300 W Xe-lamp (HAL-320, Asahi Spectra Co., Ltd.). The incident light intensity was 100 mW·cm^−2^ and the sample illumination area 0.28 cm^2^. Linear sweep voltammetry (LSV) was carried out under both dark and illumination conditions with a scan rate of 5 mV·s^−1^ with chopped light irradiation (frequency = 0.2 Hz). Stability tests were conducted by chronoamperometry under a potential of ‒0.2 V vs Ag/AgCl in a 0.5 M Na_2_SO_4_ solution. Mott-Schottky measurements were conducted using the same equipment and configuration with Mott-Schottky mode in 0.5 M Na_2_SO_4_ electrolyte with a frequency of 300 Hz in the potential range of chemical stability. Cyclic voltammetry (CV) was carried out using the same equipment and configuration in 0.5 M Na_2_SO_4_ electrolyte at the potential window 0.3 V to −0.4 V *vs* Ag/AgCl with scan rate of 100 mV S^−1^.

### Electrochemical impedance spectroscopy

Electrochemical impedance spectroscopy (EIS) was conducted using the same equipment and configuration as for the PEC measurements, which is a PCI4/300™ potentiostat equipped with EIS300™ software (Gamry Instruments, Inc.). Potentiostatic mode was applied under white light illumination (AM1.5, 100 mW·cm^−2^) at an applied voltage of −0.2 V vs. Ag/AgCl. AC perturbations of amplitude 5 mV were superimposed with frequency in the range 0.01–100 kHz. Equivalent circuit modeling and curve fitting were performed using the Echem Analyst™ software (Gamry Instruments, Inc.)

### Hydrogen production

The amount of hydrogen generated was measured using a well-sealed glass cell (100 ml) mounted with a quartz window. The Cu_2_O and Cu_2_O/NiFe-LDH working electrodes (sample illumination area = 1 cm^2^) were exposed to the light of a solar simulator equipped with a 300 W Xe-lamp (HAL-320, Asahi Spectra Co., Ltd.), and the incident AM 1.5 light intensity was 100 mW·cm^−2^. The working electrode, Pt counter-electrode and Ag/AgCl reference electrode were suspended in a solution (pH = 7) containing 30 ml of 0.1 M Na_2_SO_4_ and 10 ml of methanol (25%). Prior to testing, the reactor was repeatedly vacuum-pumped and purged with argon to remove the residual air. Then, an external bias is applied on the working electrode and the lamp is turned on. The amount of generated H_2_ gas was quantitatively analyzed by a gas chromatograph (Shimadzu GC-2014; molecular sieve 5 Å, TCD detector, Ar carrier gas).

## Additional Information

**How to cite this article**: Qi, H. *et al*. Cu_2_O Photocathode for Low Bias Photoelectrochemical Water Splitting Enabled by NiFe-Layered Double Hydroxide Co-Catalyst. *Sci. Rep.* **6**, 30882; doi: 10.1038/srep30882 (2016).

## Supplementary Material

Supplementary Information
