# Supplementary material for: Cu2O Photocathode for Low Bias Photoelectrochemical Water Splitting Enabled by NiFe-Layered Double Hydroxide Co-Catalyst
Source: Sci Rep. 2016 Aug 4;6:30882. doi: 10.1038/srep30882 (PMC4973245; doi:10.1038/srep30882)
Supplement: Supplementary Information [file srep30882-s1.pdf]

Supporting Information (SI)

Cu_2O Photocathode for Low Bias Photoelectrochemical Water Splitting Enabled by NiFe-Layered Double Hydroxide Co-Catalyst

Huan Qi,[1†] Jonathan Wolfe,[2†] Denis Fichou,[*3,4,5] and Zhong Chen.[*1]

[1] School of Materials Science Engineering, Nanyang Technological University, Singapore 639798, Singapore. Tel: +65 67904256; E-mail: aszchen@ntu.edu.sg
[2] Interdisciplinary Graduate School, Nanyang Technological University, Singapore 639798, Singapore.
[3] School of Physical and Mathematical Sciences, Nanyang Technological University, 637371, Singapore.
[4] CNRS, UMR 8232, Institut Parisien de Chimie Moléculaire, Paris, France
[5] Sorbonne Universités, UPMC Univ Paris 06, UMR 8232, Institut Parisien de Chimie Moléculaire, F-75005, Paris, France. Tel: +33 144275080; E-mail: denis.fichou@upmc.fr

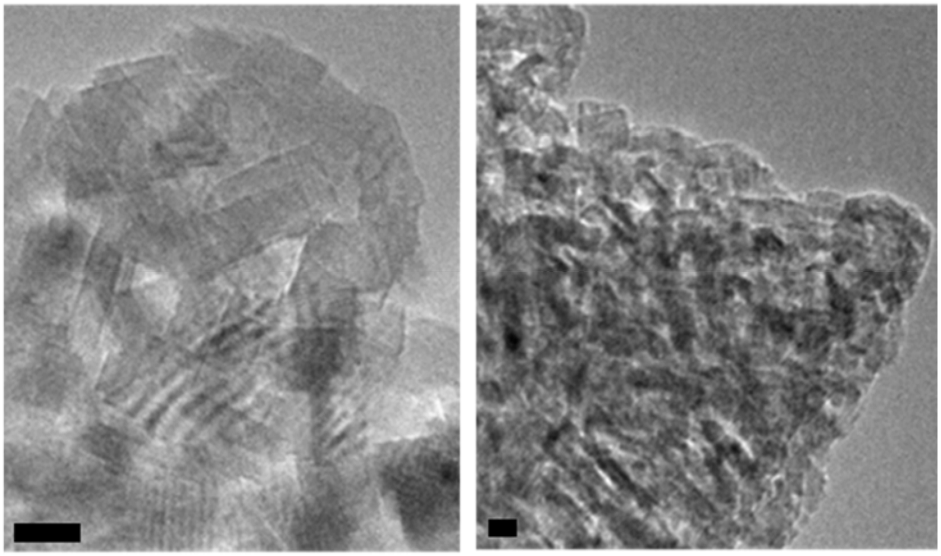

Figure S1 *TEM images of NiFe-LDH (60 s). The black scale bars represent 5 nm in both images*.

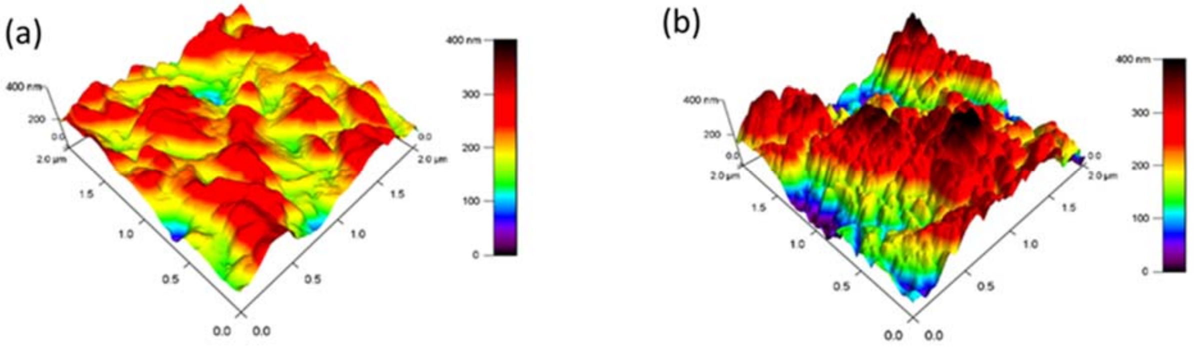

Figure S2 *AFM images of (a) bare Cu_2O and (b) Cu_2O/NiFe-LDH (20 s).*

	FTO/glass	Cu_2O	Cu_2O/NiFe-LDH (20 s)	Cu_2O/NiFe-LDH (40 s)	Cu_2O/NiFe-LDH (60 s)	Cu_2O/NiFe-LDH (150 s)
Roughness (nm)	0	45.1	47.0	53.8	66.1	80.7
Surface area (µm^2)	4	4.9	4.9	5.0	5.1	5.2

Table S1 *Roughness and surface area of our Cu_2O/NiFe-LDH samples at various deposition times based on the AFM images.*

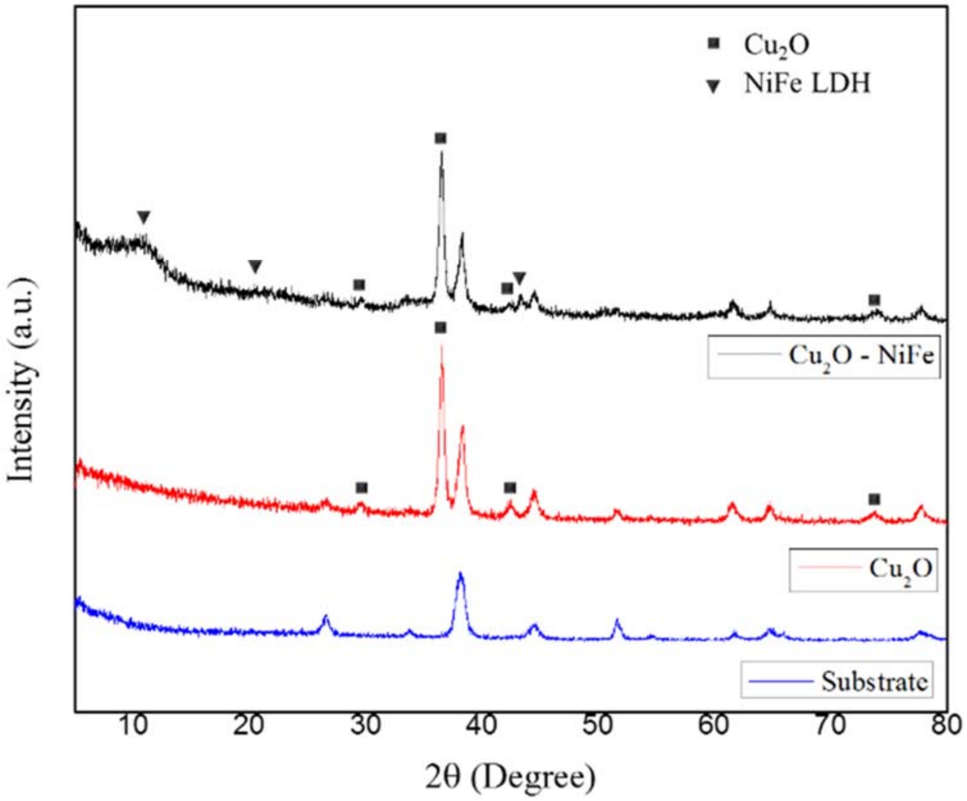

Figure S3 *XRD spectra of the glass/FTO substrate (blue curve), bare Cu_2O (red curve) and Cu_2O/NiFe-LDH (150 s) (black curve).*

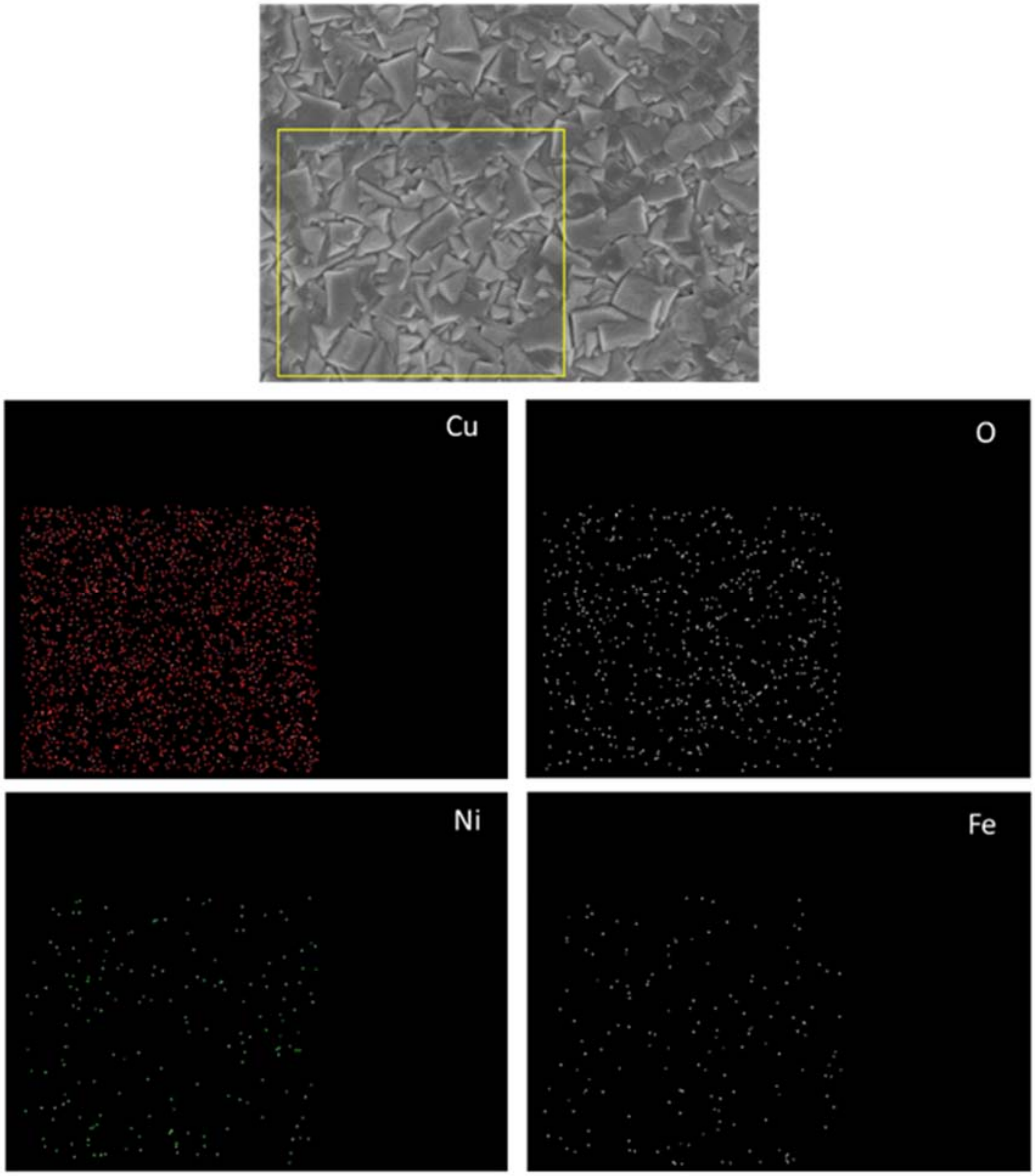

Figure S4 *Energy dispersive X-ray spectroscopy (EDX) of a typical* Cu_2O*/NiFe-LDH (20 s) sample. The area under study is indicated by a yellow square on the morphology FESEM image (top). The elements that have been tested are Cu (upper left, red spots), O (upper right, white spots), Ni (lower left, green spots) and Fe (lower right, white spots). The images reveal the low density of Ni and Fe atoms detected as compared to O and Cu, which is consistent with the reduced thickness of the NiFe-LDH overlayer after only 20 s of electrochemical deposition.*

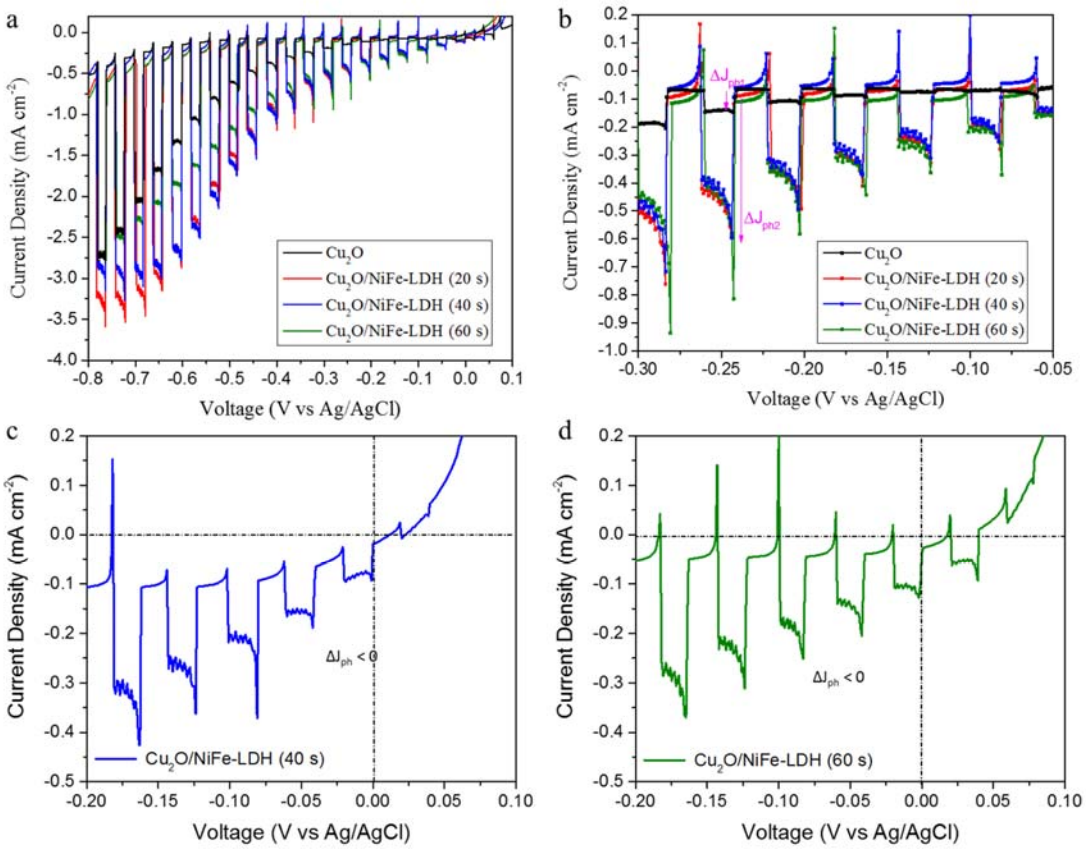

Figure S5 *Light-chopped linear sweep voltametry of Cu_2O with and without NiFe-LDH (a) under full bias range; (b) under –0.3 V to –0.05 V vs Ag/AgCl. (c) Cu_2O/NiFe-LDH (40 s) and (d) Cu_2O/NiFe-LDH (60 s) under –0.2 V to +0.1 V vs Ag/AgCl.*

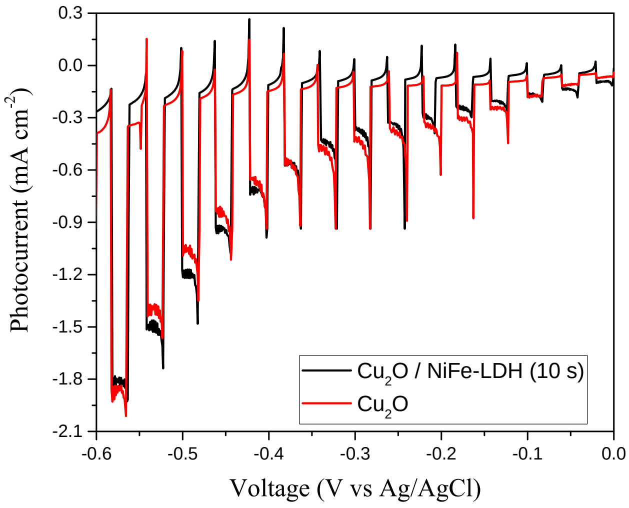

Figure S6 *Light-chopped linear sweep voltametry of bare* Cu_2O *and* Cu_2O*/NiFe-LDH (10 s)*

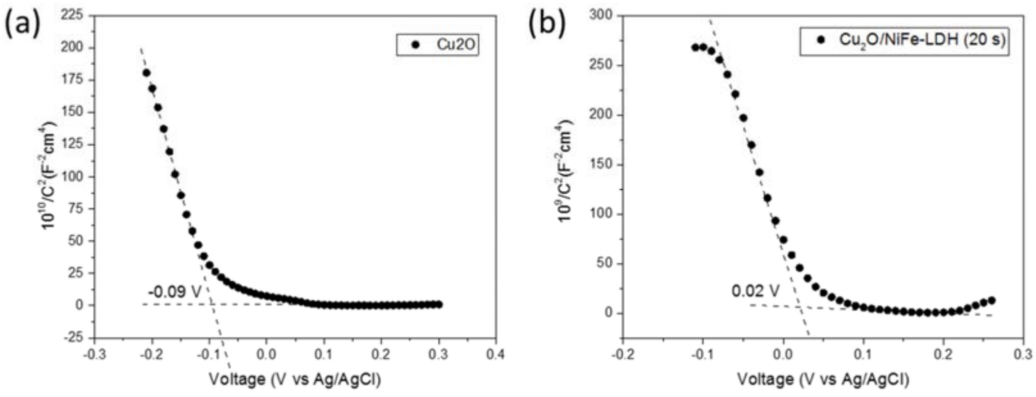

Figure S7 *Mott-Schottky plots of (a)* Cu_2O *photocathode and (b)* Cu_2O*/NiFe-LDH (20 s) photocathode.*

Figure S7 shows that Cu_2O is p-type semiconductor, with flat band potential V_{fb} -0.09 V. This value is more negative than the reported flat band potentials (0.7 vs RHE).[1] After deposit with 20 s NiFe-LDH, the Cu_2O/NiFe-LDH photocathode still shows p-type but V_{fb} value raised up from -0.09V to 0.02V. According to Eq 1, the calculated charge carrier number N_D for pure Cu_2O is 1.9×10^{19} cm^{-3} and the calculated charge carrier number N_D for Cu_2O/NiFe-LDH (20 s) is 9×10^{19} cm^{-3}. This increased number of carriers that has reached the electrode/electrolyte interface is the results of increase in charge separation brought by NiFe-LDH.

$$N_D = \frac{2}{e\varepsilon\varepsilon_0 k_{MS}} \qquad (1)$$

where wherein ε_0 is the permittivity of vacuum, ε is the relative permittivity of Cu_2O (~7.5), k_{MS} is the slop.

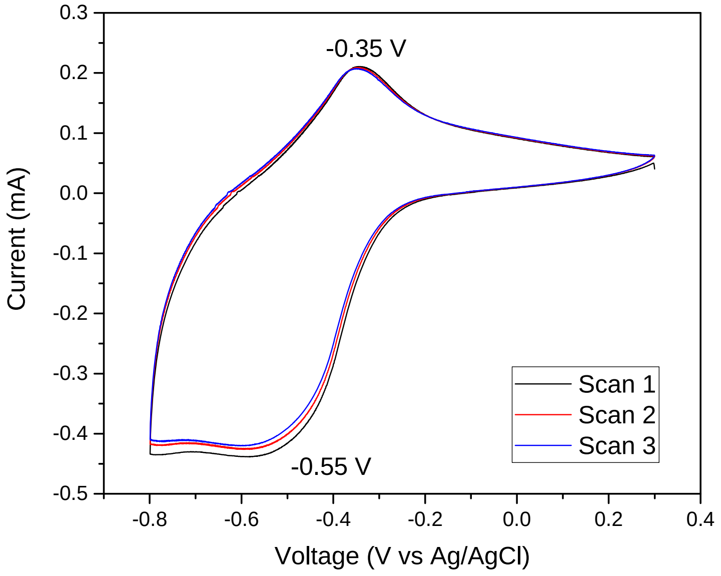

Figure S8 *Cyclic voltammetry of NiFe-LDH (60 s)*

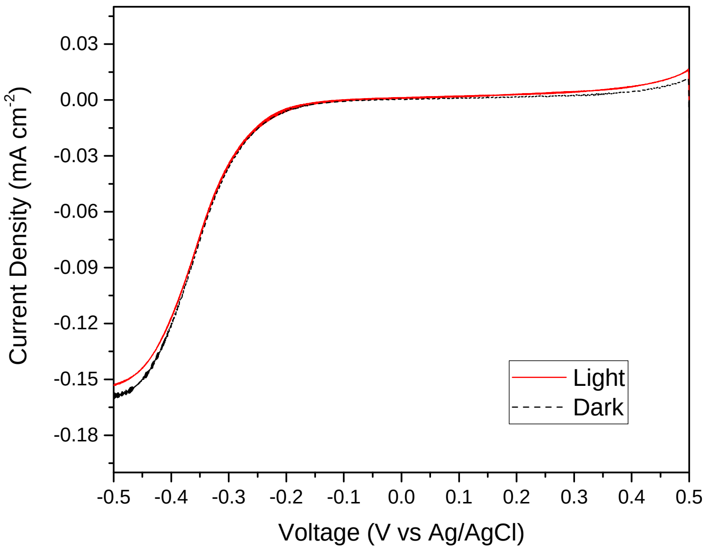

Figure S9 *Photoresponse of NiFe-LDH (300 s)*

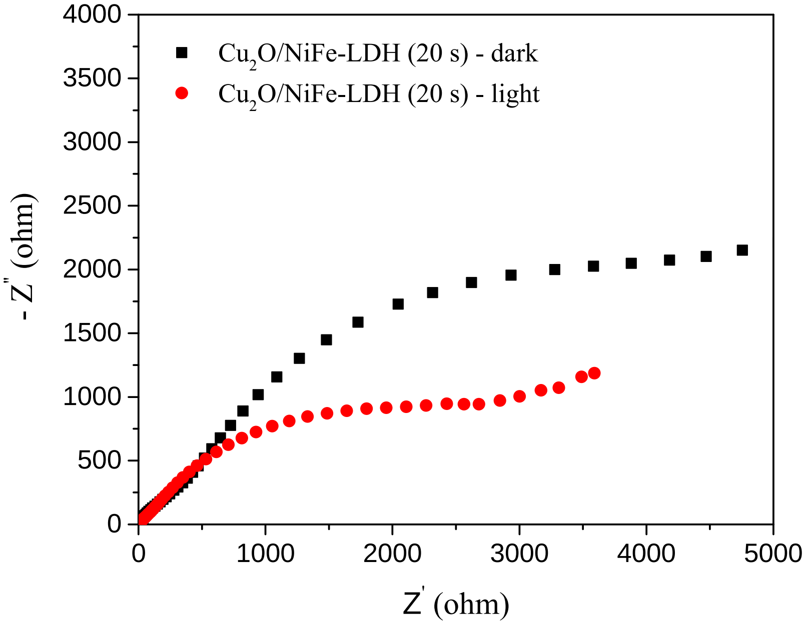

Figure S10 *Electrochemical impedance spectroscopy (EIS) of Cu_2O/NiFe-LDH (20 s) in the dark and under illumination (AM1.5, 100 mW cm^{-2}) at a –0.2 V vs Ag/AgCl bias.*

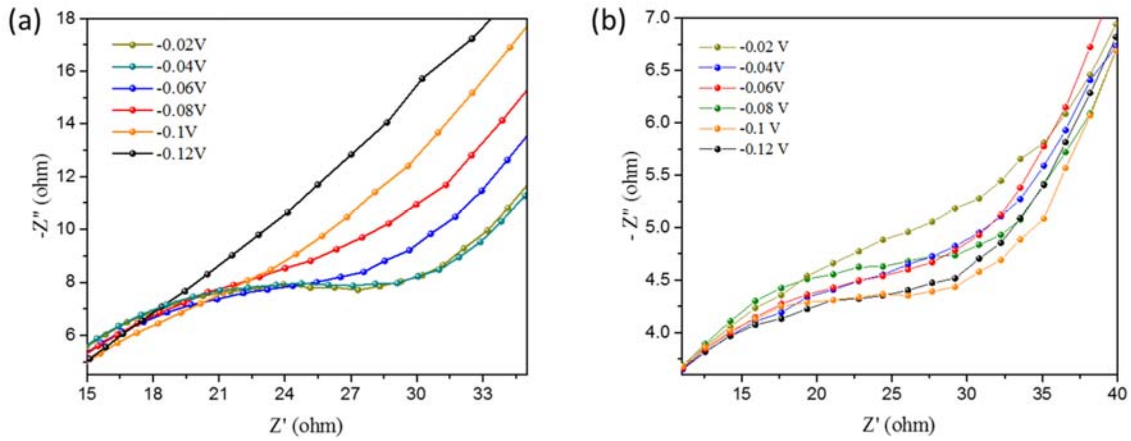

Figure S11 Enlarged EIS of (a) Cu_2O and (b) Cu_2O/NiFe-LDH under different bias.

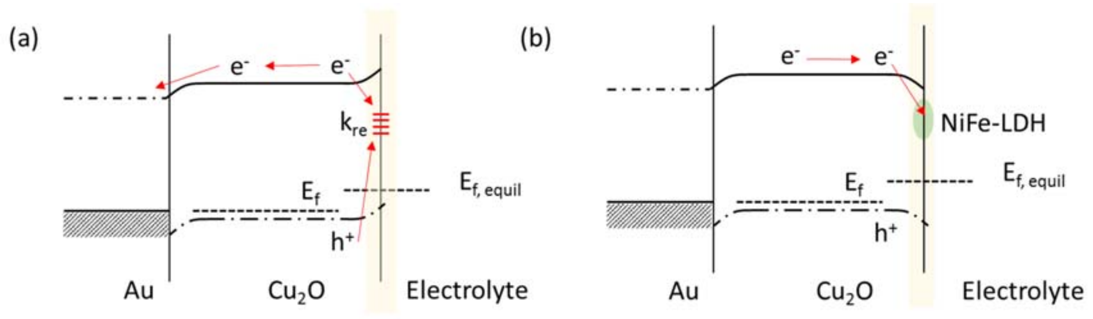

Figure S12. *Proposed charge recombination and charge transportation processes of (a) Cu_2O photocathode and (b) Cu_2O/NiFe-LDH photocathode.*

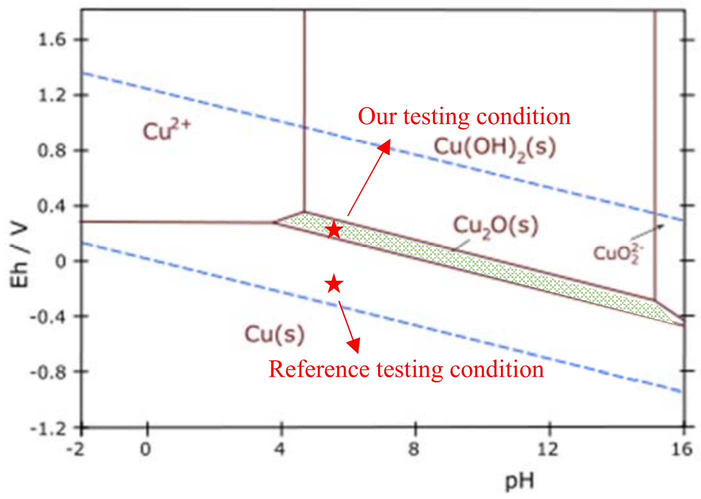

Figure S13. *Pourbaix diagram for copper.*[2]

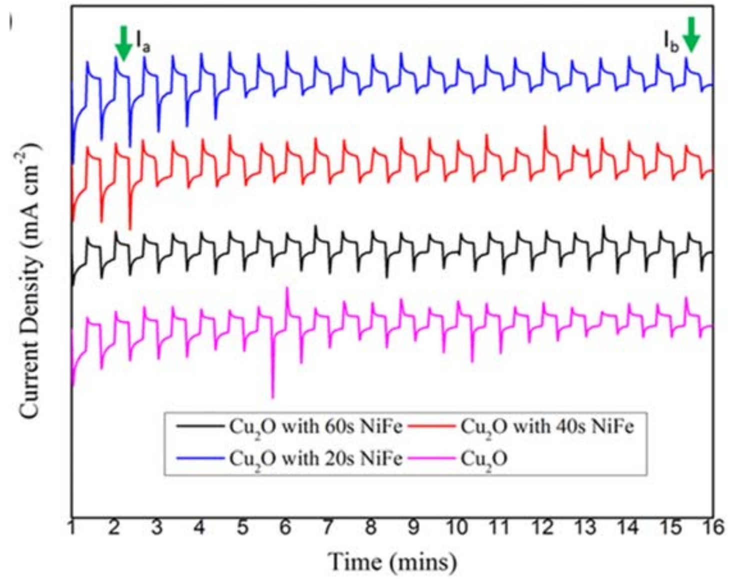

Sample	I_b/I_a
Cu_2O	53%
Cu_2O / 20s NiFe	67%
Cu_2O / 40s NiFe	79%
Cu_2O / 60s NiFe	81%

Figure S14 *Stability test of bare Cu_2O and Cu_2O/NiFe-LDH for various deposition times (20, 40 and 60 s) recorded under –0.6 V vs Ag/AgCl. The table on the right provides the residual current ratio I_b/I_a (indicated by the green arrows) obtained after 15 minutes of illumination.*

1 Li, C. L., Li, Y. B. & Delaunay, J. J. A Novel Method to Synthesize Highly Photoactive Cu2O Microcrystalline Films for Use in Photoelectrochemical Cells. *Acs Appl Mater Inter* **6**, 480-486, doi:10.1021/am404527q (2014).

2 Pourbaix,M - Atlas of Electrochemical Equilibria in Aqueous Solutions. *Anti-Corros* **14**, 28-& (1967).
